# Systems biology approach reveals that overflow metabolism of acetate in *Escherichia coli *is triggered by carbon catabolite repression of acetyl-CoA synthetase

**DOI:** 10.1186/1752-0509-4-166

**Published:** 2010-12-01

**Authors:** Kaspar Valgepea, Kaarel Adamberg, Ranno Nahku, Petri-Jaan Lahtvee, Liisa Arike, Raivo Vilu

**Affiliations:** 1 Tallinn University of Technology, Department of Chemistry, Akadeemia tee 15, 12618 Tallinn, Estonia; 2 Competence Centre of Food and Fermentation Technologies, Akadeemia tee 15b, 12618 Tallinn, Estonia; 3 Tallinn University of Technology, Department of Food Processing, Ehitajate tee 5, 19086 Tallinn, Estonia

## Abstract

**Background:**

The biotechnology industry has extensively exploited *Escherichia coli *for producing recombinant proteins, biofuels etc. However, high growth rate aerobic *E. coli *cultivations are accompanied by acetate excretion *i.e*. overflow metabolism which is harmful as it inhibits growth, diverts valuable carbon from biomass formation and is detrimental for target product synthesis. Although overflow metabolism has been studied for decades, its regulation mechanisms still remain unclear.

**Results:**

In the current work, growth rate dependent acetate overflow metabolism of *E. coli *was continuously monitored using advanced continuous cultivation methods (A-stat and D-stat). The first step in acetate overflow switch (at μ = 0.27 ± 0.02 h^-1^) is the repression of acetyl-CoA synthethase (Acs) activity triggered by carbon catabolite repression resulting in decreased assimilation of acetate produced by phosphotransacetylase (Pta), and disruption of the PTA-ACS node. This was indicated by acetate synthesis pathways PTA-ACKA and POXB component expression down-regulation before the overflow switch at μ = 0.27 ± 0.02 h^-1 ^with concurrent 5-fold stronger repression of acetate-consuming Acs. This in turn suggests insufficient Acs activity for consuming all the acetate produced by Pta, leading to disruption of the acetate cycling process in PTA-ACS node where constant acetyl phosphate or acetate regeneration is essential for *E. coli *chemotaxis, proteolysis, pathogenesis etc. regulation. In addition, two-substrate A-stat and D-stat experiments showed that acetate consumption capability of *E. coli *decreased drastically, just as Acs expression, before the start of overflow metabolism. The second step in overflow switch is the sharp decline in cAMP production at μ = 0.45 h^-1 ^leading to total Acs inhibition and fast accumulation of acetate.

**Conclusion:**

This study is an example of how a systems biology approach allowed to propose a new regulation mechanism for overflow metabolism in *E. coli *shown by proteomic, transcriptomic and metabolomic levels coupled to two-phase acetate accumulation: acetate overflow metabolism in *E. coli *is triggered by Acs down-regulation resulting in decreased assimilation of acetic acid produced by Pta, and disruption of the PTA-ACS node.

## Background

*Escherichia coli *has not only been the prime organism for developing new molecular biology methods but also for producing recombinant proteins, low molecular weight compounds etc. in industrial biotechnology for decades due to its low cost manufacturing and end-product purification and its ability to reach high cell densities grown aerobically [1,2]. However, a major problem exists with aerobic *E. coli *cultivation on glucose at high growth rates-formation and accumulation of considerable amounts of acetic acid *i.e*. overflow metabolism. In addition to being detrimental for target product synthesis, accumulated acetate inhibits growth and diverts valuable carbon from biomass formation [3,4].

**Figure 1:**
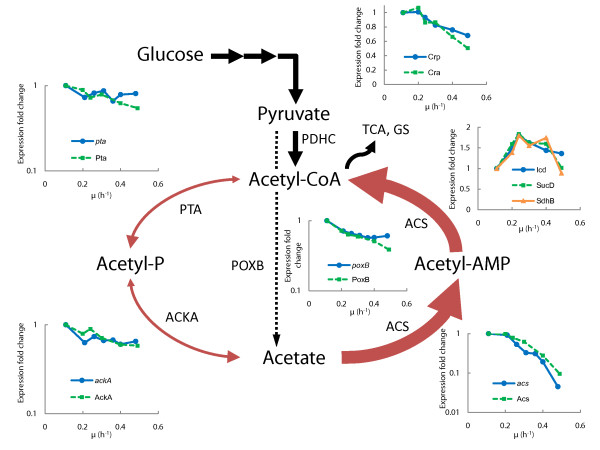
**Effect of specific growth rate on acetate synthesis and utilization pathways, selected TCA cycle and carbon catabolite repressed gene and protein expression levels in *E. coli *A-stat experiments**. PTA, phosphotransacetylase; ACKA, acetate kinase; ACS, acetyl-CoA synthetase; POXB, pyruvate oxidase; PDHC, pyruvate dehydrogenase complex; TCA, tricarboxylic acid cycle; GS, glyoxylate shunt; Crp, cyclic AMP receptor protein; Cra, catabolite repressor activator; Icd, isocitrate dehydrogenase; SucD, succinyl-CoA synthethase; SdhB, succinate dehydrogenase; μ, specific growth rate (h^-1^). Thickness of red arrows denotes level of ACS and PTA-ACKA pathway repression (thick line represents stronger repression). Protein data points are average of two independent experiments, error bars are not shown for better visualization. Gene names are italicized. Refer to Additional file 2 for standard deviations and all the data.

The acetate synthesis and utilization pathways [5] can be seen in Figure 1: acetate can be synthesized by phosphotransacetylase (PTA)/acetate kinase (ACKA) and by pyruvate oxidase (POXB). Acetic acid can be metabolized to acetyl-CoA either by the PTA-ACKA pathway or by acetyl-CoA synthetase (ACS) through an intermediate acetyl-AMP. The high affinity (*K*_m _of 200 μM for acetic acid) ACS scavenges acetate at low concentrations whereas the low affinity PTA-ACKA pathway (*K*_m _of 7-10 mM) is activated in the presence of high acetate concentrations [6].

The phenomenon of overflow metabolism has been studied widely over the years and it is commonly believed to be caused by an imbalance between the fluxes of glucose uptake and those for energy production and biosynthesis [7,8]. Several explanations such as the saturation of catalytic activities in the tricarboxylic acid (TCA) cycle [9,10] and respiratory chain [7,11,12], energy generation [5,13] or the necessity for coenzyme A replenishment [14] have been proposed. In addition to bioprocess level approaches [1,15], various genetic modifications of the acetate synthesis pathways extensively reviewed in De Mey *et al*. [15] have been made to minimize acetic acid production. For instance, it has been shown that deleting the main acetate synthesis route PTA-ACKA results in a strong reduction (up to 80%) of acetate excretion, maximum growth rate (*ca *20%) and elevated levels of formate and lactate (*ca *30-fold) [4,16-18], whereas *poxB *disruption causes reduction in biomass yield (*ca *25%) and loss of aerobic growth efficiency of *E. coli *[19]. The latter indicates that acetate excretion cannot be simply excluded by disrupting its synthesis routes without encountering other unwanted effects. Unfortunately, no clear conclusions could be drawn from batch experiments with an *acs *knock-out strain [4]. It should be noted that studies with *E. coli *genetically modified strains engineered to diminish acetate production in batch cultures have not fully succeeded in avoiding acetate accumulation together with increasing target product production yields and rates [15]. Additionally, these studies have not allowed elucidating the mechanism of overflow metabolism unequivocally [4,20,21].

Acetate overflow is a growth rate dependent phenomenon, but no study has specifically focused on growth rate dependency of protein and gene expression regulation, intra-and extracellular metabolite levels using also metabolic modeling. Describing the physiology of an organism on several 'omic levels is the basis of systems biology that facilitates better understanding of metabolic regulation [22]. In this study, *E. coli *metabolism at proteomic, transcriptomic and metabolomic levels was investigated using continuous cultivation methods prior to and after overflow metabolism was switched on. Usually, chemostat cultures are used for steady state metabolism analysis, however, we applied two changestat cultivation techniques: accelerostat (A-stat) and dilution rate stat (D-stat), see Methods section for details [23,24]. These cultivation methods were used as they provide three advantages over chemostat. Firstly, these changestat cultivation techniques precisely detect metabolically relevant switch points (*e.g*. start of overflow metabolism, maximum specific growth rate) and enable to monitor the dynamic patterns of several metabolic physiological responses simultaneously which could be left unnoticed using chemostat. Secondly, it is possible to collect vast amount of steady state comparable samples and by doing so, save time. Thirdly, both A-stat and D-stat enable to quantitatively study specific growth rate dependent co-utilization of growth substrates. Latter advantage was applied for investigating acetic acid consumption capability of *E. coli *at various dilution rates in this study. Combining changestat cultivation methods enables to study metabolism responses of the same genotype at different physiological states in detail without encountering the possible metabolic artifacts accompanied when using genetically modified strains.

Results obtained by studying specific growth rate dependent changes in *E. coli *proteome, transcriptome and metabolome in continuous cultures together with metabolic modeling allowed us to propose a new theory for acetate overflow: acetate excretion in *E. coli *is triggered by carbon catabolite repression mediated down-regulation of Acs resulting in decreased assimilation of acetate produced by Pta, and disruption of the PTA-ACS node.

## Results

### *E. coli *metabolic switch points characterization

**Figure 2:**
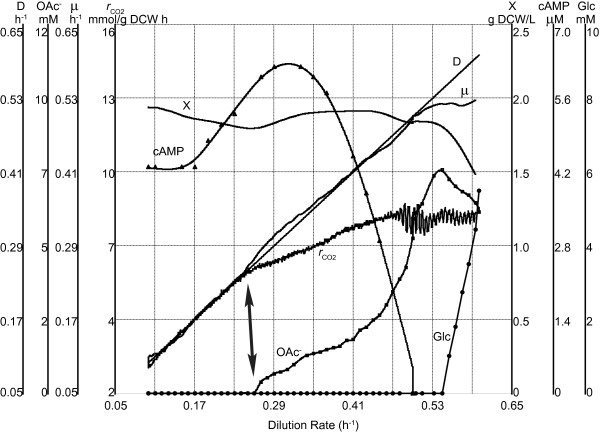
**Increasing dilution rate dependent *E. coli *metabolism characterization in one A-stat cultivation (a = 0.01 h^-2^)**. D, dilution rate (h^-1^); X, biomass concentration (g dry cellular weight (DCW)/L); μ, specific growth rate (h^-1^); *r*_CO2_, specific CO_2 _production rate (mmol/g DCW h); OAc^-^, acetate concentration (mM); Glc, glucose concentration (mM); cAMP, cyclic AMP concentration (μM). Arrow indicates the start of overflow metabolism. Start of vertical axes was chosen for better visualization.

In all accelerostat (A-stat) cultivation experiments, after the culture had been stabilized in chemostat at 0.10 h^-1 ^to achieve steady state conditions, continuous increase in dilution rate with acceleration rate (a) 0.01 h^-2 ^(0.01 h^-1 ^per hour) was started. Continuous change of specific growth rate resulted in detecting several important changes in *E. coli *metabolism as demonstrated in Figure 2. Firstly, in A-stat cultivations where glucose was the only carbon source in the medium, acetic acid started to accumulate (*i.e*. overflow metabolism switch) at μ = 0.27 ± 0.02 h^-1 ^(average ± standard deviation) and a two-phase acetate accumulation pattern was observed (discussed below; Figure 2). Cells reached maximum CO_2 _production and O_2 _consumption at μ = 0.46 ± 0.02 h^-1 ^and metabolic fluctuations were observed at μ = 0.49 ± 0.03 h^-1 ^followed by washout of culture at μ = 0.54 ± 0.03 h^-1 ^(corresponding to maximum specific growth rate at given conditions). The nature of these fluctuations will be studied further and not covered in the current publication. All A-stat results were reproduced with relative standard deviation less than 10% with the exception of acetate production per biomass (Y_OAc-_) (Table 1 and Figure S1 in Additional file 1).

**Table 1:** A-stat and chemostat growth characteristics comparison and A-stat reproducibility over the studied specific growth rate range for three independent experiments.

	μ = 0.24 h^-1^		μ = 0.30 h^-1^		μ = 0.40 h^-1^		μ = 0.51 h^-1^		μ = 0.10-0.47 h^-1^
	**Chemostat**	**A-stat**	**Chemostat**	**A-stat**	**Chemostat**	**A-stat**	**Chemostat**	**A-stat**	**A-stat RSD, %**
	
Y_XS_^a^	0.44	0.40 ± 0.01	0.46	0.41 ± 0.01	0.44	0.42 ± 0.00	0.43	0.41 ± 0.01	2.0
Y_OAc-_^b^	NDE	NDE	0.53	0.90 ± 0.32	1.70	1.56 ± 0.23	3.25	3.35 ± 0.82	ND
Y_cAMP_^c^	3.47	3.59 ± 0.39	3.25	3.55 ± 0.32	2.70	2.17 ± 0.07	0.86	0.71^e^	9.1
Y_CO2_^d^	27.56	30.12 ± 2.04	27.55	27.19 ± 1.22	26.24	23.86 ± 1.41	ND	21.19 ± 0.19	5.6

A-stat values represent the average from three independent experiments and standard deviation follows the ± sign. Chemostat values from one experiment. NDE, not detected. ND, not determined. RSD, relative standard deviation.

^a^Biomass yield is given in g dry cell weight (DCW)/g glucose consumed (g DCW/g glucose).

^b^Acetic acid production per biomass is given in mmol acetic acid/g DCW.

^c^cAMP production per biomass is given in μmol cAMP/g DCW.

^d^Carbon dioxide (CO_2_) production per biomass is given in mmol CO_2_/g DCW.

^e^Data from one A-stat experiment.

### Metabolomic responses to rising specific growth rate

A-stat cultivation enabled to study acetic acid accumulation profile in detail with increasing specific growth rate. Interestingly, a two-phase acetate accumulation pattern was observed (Figure 2). Slow accumulation of acetic acid started at μ = 0.27 ± 0.02 h^-1 ^with concomitant change in specific CO_2 _production rate (Figure 2). Faster accumulation of acetate was witnessed after cells had reached maximum CO_2 _production at μ = 0.46 ± 0.02 h^-1^. Quite surprisingly, production of the important carbon catabolite repression (CCR) signal molecule cAMP (Y_cAMP_) rose from steady state chemostat level 2.45 ± 0.26 μmol/g dry cellular weight (DCW) (μ = 0.10 h^-1^) to 3.55 ± 0.32 μmol/g DCW (μ = 0.30 h^-1^) after which it sharply decreased to 1.30 ± 0.44 μmol/g DCW at μ = 0.45 h^-1 ^(Figure S1 in Additional file 1). This abrupt decline took place simultaneously with the faster acetate accumulation profile described above (Figure 2 and Figure S1 in Additional file 1). In addition, similar two-phase acetate accumulation phenomenon was observed in a two-substrate (glucose + acetic acid) A-stat during the decrease of cAMP around specific growth rate 0.39 h^-1 ^(Figure S2 in Additional file 1).

**Figure 3:**
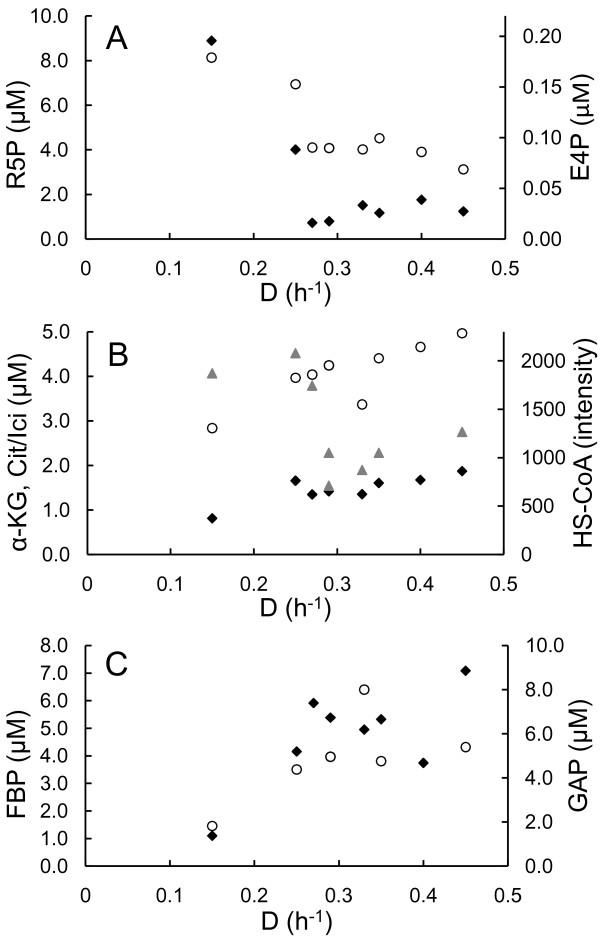
**Dilution rate dependent intracellular metabolite patterns in one *E. coli *A-stat experiment**. D, dilution rate (h^-1^). (A) Pentose phosphate pathway metabolites. R5P, ribose-5-phosphate concentration (black diamond); E4P, erythrose-4-phosphate concentration (open circle). (B) TCA cycle metabolites and co-factor free CoA. α-KG, α-ketoglutarate concentration (black diamond); Cit/Ici, citrate/isocitrate pool concentration (open circle); HS-CoA, co-factor free CoA level (grey triangle). (C) Glycolysis (upper part) metabolites. FBP, fructose-1,6-bisphosphate concentration (black diamond); GAP, glyceraldehyde-3-phosphate concentration (open circle).

Significant fall in two of the measured pentose phosphate pathway intermediates ribose-5-phosphate (R5P) and erythrose-4-phosphate (E4P) was detected with increasing specific growth rate which could point to possible limitation in RNA biosynthesis during growth (Figure 3A). PTA-ACS node related compound nonesterified acetyl-CoA (HS-CoA) level declined two-fold simultaneously with cAMP after acetate started to accumulate (Figure 3B). This indicates the possible increase of other CoA containing compounds *e.g*. succinyl-CoA. Accumulation of TCA cycle intermediates α-ketoglutarate and isocitrate (Figure 3B) with increasing dilution rate could be associated with pyrimidine deficiency and decrease of ATP expenditure in the PTA-ACS cycle. Concurrently, intracellular concentrations of fructose-1,6-bisphosphate (FBP) and glyceraldehyde-3-phosphate (GAP) from the upper part of energy generating glycolysis increased 6- and 3-fold, respectively (Figure 3C).

### Functional-genomic responses to rising specific growth rate

The two main known pathways for acetate synthesis phosphotransacetylase-acetate kinase (PTA-ACKA) and pyruvate oxidase (POXB) were down-regulated, both on gene and protein expression levels, from μ = 0.20 h^-1 ^*i.e*. before acetate overflow was switched on. At the same time, there was a concurrent 10-fold repression of the acetic acid utilization enzyme acetyl-CoA synthetase (Acs). This substantial difference (5-fold) between the acetate synthesis and assimilation pathways expression suggests that the synthesized acetic acid cannot be fully assimilated with increasing growth rates (Figure 1).

**Figure 4:**
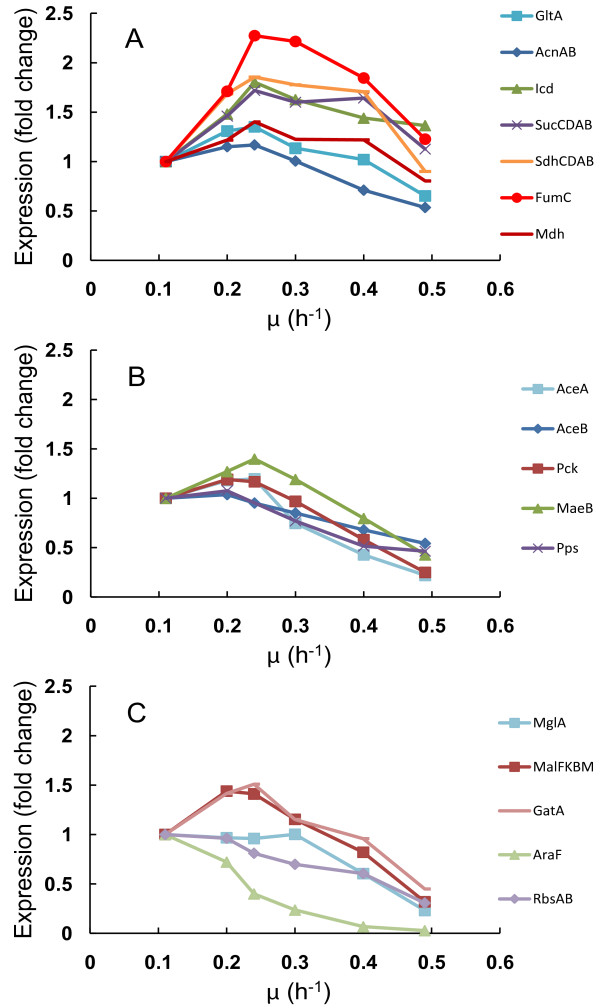
**Specific growth rate dependent TCA cycle, glyoxylate shunt, glyconeogenesis and carbon catabolite repressed protein expression changes in *E coli *A-stat cultures**. μ, specific growth rate (h^-1^). (A) TCA cycle (average of proteins from the same operon are depicted as one point *e.g*. AcnAB). (B) Glyoxylate shunt (AceA, AceB) and glyconeogenesis. (C) Carbon catabolite repressed proteins. Protein data points are average of two independent experiments, error bars are not shown for better visualization (refer to Additional file 2 for standard deviations).

We observed the beginning of carbon catabolite repression (CCR) induction prior to acetate accumulation in parallel with Acs down-regulation. This was indicated by down-regulation (3-fold on average) of CCR-mediated components: alternative (to glucose) substrate transport and utilization systems like galactose (MglAB), maltose (MalBEFKM), galactitol (GatABC), L-arabinose (AraF), D-ribose (RbsAB), C_4_-dicarboxylates (DctA) and acetate (ActP, YjcH) (Figure 4C and Additional file 2). Moreover, expression of transcription activator Crp (cyclic AMP receptor protein which regulates the expression of Acs transcribing *acs-yjcH-actP *operon) and Cra (catabolite repressor activator; a global transcriptional protein essential for acetic acid uptake [25]) were reduced 1.5 and 2 times, respectively, in like manner to carbon catabolite repressed proteins mentioned above (Figure 1). Simultaneously, components of the gluconeogenesis pathway (Pck, MaeB, Pps) and glyoxylate shunt enzymes AceA, AceB (vital for acetate consumption) were repressed with growth rate increase (Figure 4B and Additional file 2). It should be emphasized that most of the TCA cycle gene and protein levels were maintained or even increased up to μ = 0.40 h^-1 ^followed by sudden repression simultaneous to achieving maximum specific CO_2 _production rate (μ = 0.46 ± 0.02 h^-1^, see above; Figure 1 Figure 2 and Figure 4A). This may allude to no limitation at the TCA cycle level around the specific growth rate where overflow metabolism was switched on.

### Acetic acid consumption capability studied by dilution rate stat (D-stat) and two-substrate A-stat cultivations

**Figure 5:**
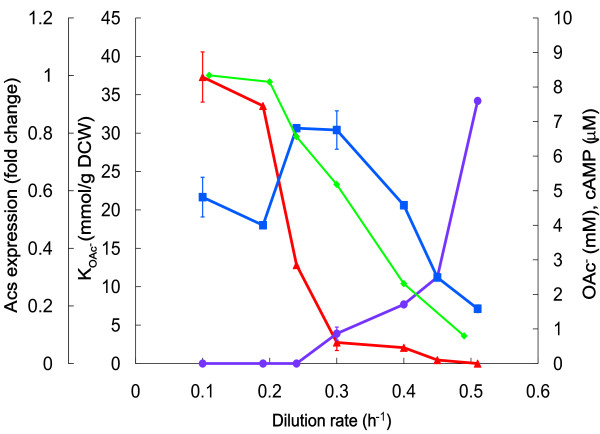
**Dilution rate dependent acetic acid consumption capability in *E. coli *D-stat cultures**. K_OAc_-, acetic acid consumption per biomass (red triangle); OAc^-^, acetate concentration in chemostat before the start of acetic acid supplemented medium addition (violet circle); cAMP, cyclic AMP concentration (blue square); Acs, acetyl-CoA synthetase protein dilution rate dependent expression levels from A-stat (green diamond). Error bars represent the standard deviation from two independent D-stat experiments.

The beginning of a strong decrease in acetate assimilation enzyme Acs expression before overflow switch point implies to a possible connection between acetate assimilation capability and overflow metabolism of acetate (Figure 1). Therefore, specific growth rate dependent acetic acid consumption capabilities were investigated using D-stat and two-substrate A-stat methods. It was shown by D-stat experiments at various dilution rates that more than a 12-fold reduction in acetate consumption capability took place around overflow switch point, and its total loss was detected between dilution rates 0.45 and 0.505 ± 0.005 h^-1 ^(Figure 5). Acetic acid consumption and production was also studied in a single experiment using two substrate (glucose + acetic acid) A-stat cultivation (Figure S2 in Additional file 1) which demonstrated that acetic acid consumption started to decrease at μ = 0.25 h^-1 ^and was completely abolished at μ = 0.48 h^-1 ^which fits into the range of dilution rates observed in D-stat.

### A-stat comparison with chemostat

As could be seen from Table 1 major growth characteristics such as biomass yield (Y_XS_), acetate (Y_OAc-_), cyclic AMP (Y_cAMP_) and carbon dioxide (Y_CO2_) production per biomass from A-stat and chemostat are all fully quantitatively comparable. The latter results enable to use A-stat data for quantitative modeling calculations. In addition, the two continuous cultivation methods were examined at transcriptome level using DNA microarrays. Transcript spot intensities from quasi steady state A-stat sample at μ = 0.48 h^-1 ^and chemostat sample at μ = 0.51 h^-1 ^showed an excellent Pearson product-moment correlation coefficient R = 0.964 (Figure S3 in Additional file 1; Additional file 3). This indicates good biological correlation between *E. coli *transcript profiles at similar specific growth rates in chemostat and A-stat. These results showed that our quasi steady state data from A-stat and D-stat cultures are steady state representative.

### Proteome and transcriptome comparison

*E. coli *protein expression ratios for around 1600 proteins were generated by comparing two biological replicates at specific growth rates 0.20 ± 0.01; 0.26; 0.30 ± 0.01; 0.40 ± 0.00; 0.49 ± 0.01 h^-1 ^with sample at μ = 0.10 ± 0.01 h^-1 ^(chemostat point prior to the start of acceleration in A-stat) which produced Pearson correlation coefficients for two biological replicates in the indicated pairs of comparison in the range of R = 0.788-0.917 (Figure S4 in Additional file 1).

DNA microarray analysis of 4,321 transcripts was conducted with the Agilent platform using the samples from one A-stat cultivation. Gene expression ratios between specific growth rates 0.21; 0.26; 0.31; 0.36; 0.40; 0.48 h^-1 ^and μ = 0.11 h^-1 ^(chemostat point prior to the start of acceleration in A-stat) were calculated. Comparison of gene and protein expression changes (between respective specific growth rates) revealed that components of the PTA-ACS node were regulated at transcriptional level as the absolute majority of the studied transcripts and proteins indicated by the good correlation between transcriptome and proteome expression profiles (Figure 1 and Figure S5 in Additional file 1).

Most recent studies have either failed to find a significant correlation between protein and mRNA abundances or have observed only a weak correlation (reviewed in [22]). It has been suggested that the main reasons for uncoupling of mRNA and protein abundances are protein regulation by post-translational modification, post-transcriptional regulation of protein synthesis, differences in the half-lives of mRNA and proteins, or possible functional requirement for protein binding [22]. As the cells in these studies were mostly cultured in non steady state condition, our steady state data with very good correlation between transcriptome and proteome implies that the physiological state of the culture (steady state vs. non steady state) could be an important factor in terms of mRNA and protein correlation determination. Transcriptome and proteome data are presented in Additional file 2 and at NCBI Gene Expression Omnibus and PRIDE database (see Methods for details), respectively.

## Discussion

To gain better insights into the regulation of acetate overflow metabolism in *E. coli*, we studied specific growth rate dependent proteomic, transcriptomic and metabolomic patterns combined with metabolic modeling using advanced continuous cultivation methods, which has not been carried out before. Continuous monitoring of the specific growth rate effect on *E. coli *metabolism enabled us to precisely detect important metabolic shift points, the most important being the start of acetate overflow at μ = 0.27 ± 0.02 h^-1 ^(Figure 2), and changing patterns of a number of important metabolites *e.g*. acetate, cAMP. Quite surprising was the down-regulation of the known acetate synthesis pathways, PTA-ACKA and POXB expression before overflow switch with increasing growth rate (Figure 1). A similar pattern has been seen before in chemostat cultures but without emphasizing the possible physiological consequences [26-28]. A 10-fold repression of the acetic acid utilization enzyme acetyl-CoA synthetase (Acs) expression was observed concurrently with the down-regulation of the PTA-ACKA pathway indicating that acetic acid synthesis might exceed its assimilation (Figure 1). Our two substrate A-stat and D-stat experiments directly proved that acetate consumption capability of *E. coli *is specific growth rate dependent as acetate consumption started to decrease at μ = 0.25 h^-1 ^(Figure S2 in Additional file 1) and acetate consumption capability decreased 12-fold around overflow switch growth rate μ = 0.27 ± 0.02 h^-1^, respectively (Figure 5). In addition, it was shown that activation of carbon catabolite repression (CCR) and repression of Acs take place simultaneously prior to the start of overflow metabolism (Figure 1 Figure 4 and Figure 5). As a result, it is proposed that acetate overflow metabolism in *E. coli *is triggered by Acs down-regulation resulting in decreased assimilation of acetic acid produced by Pta, and disruption of the PTA-ACS node.

**Figure 6:**
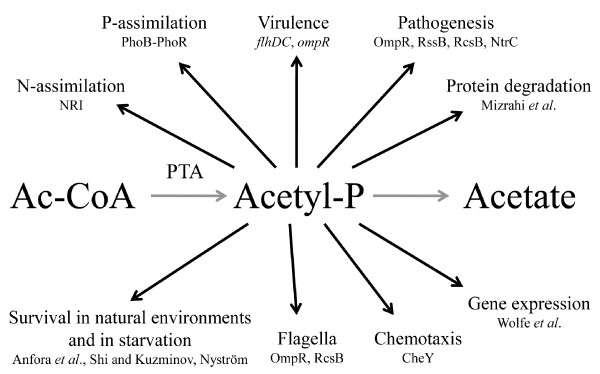
**Acetyl-P as an important signal molecule**. Ac-CoA, acetyl-CoA; PTA, phosphotransacetylase; refer to text for other abbreviations.

We showed that Acs was concurrently down-regulated five times more compared to the acetate synthesis pathways (Figure 1). In addition, the TCA cycle flux decrease as shown by change in CO_2 _production at overflow switch growth rate indicates that carbon is not metabolized by the TCA cycle after the start of acetate accumulation with pre overflow switch rates (Figure 2 and Additional file 4). The latter is caused because the amount of acetyl-CoA entering the TCA cycle decreases after carbon is lost into excreted acetate. Stronger repression of the acetate consuming Acs in comparison with acetate synthesizing PTA-ACKA together with a decline in TCA cycle flux suggest disruption of acetic acid cycling at the PTA-ACS node (Figure 1). While this node may seem as a futile cycle, the fact is that numerous metabolic tasks involving the intermediate molecules of this cycle-acetyl phosphate (acetyl-P) and acetyl-AMP-are essential for proper *E. coli *growth (Figure 6). For instance, these molecules play a crucial role in bacterial chemotaxis regulation in which flagellar rotation is controlled by the activation level of the response regulator CheY [29] through either phosphotransfer from CheA [30,31] or acetyl-P [31,32], acetylation by acetyl-AMP [33,34] or co-regulation of both mechanisms [29]. It has been also demonstrated that acetyl-P synthesis is vital for EnvZ-independent regulation of outer membrane porins [35], pathogenesis [36] and regulation of several virulence factors [5]. Furthermore, it has been presented that acetyl-P interacts with phosphate concentration regulators PhoB-PhoR [37] and NRI protein which is part of a complex nitrogen sensing system [38]. Acetyl-P is critical for efficient degradation of unfolded or damaged proteins by ATP-dependent proteases [39]. Altogether, acetyl-P can influence the regulation of almost 100 other genes [40]. Finally, *pta *and/or *ackA *mutations were shown to affect repair-deficient *E. coli *mutants [41] and a *pta *mutant has been reported to be impaired in its ability to survive during glucose starvation, while the *ackA *mutant survived as well as the parent strain [42]. It is important to note that the only known pathway in *E. coli *for acetyl-P synthesis is the PTA-ACKA [5,31]. Taking all the previous into account, we conclude that acetyl-P as well as acetyl-AMP are essential for cellular growth of *E. coli*, and as acetic acid formation is the result of their dephosphorylation, acetic acid should be synthesized and consumed simultaneously during growth to maintain proper balance between metabolites of the PTA-ACS node. This is in agreement with Shin *et al*. [28] who proposed that wild-type *E. coli *constitutively synthesizes acetate even when growing on non-acetogenic carbon source succinate or at low growth rates in carbon limited cultures. It also has to be mentioned that acetic acid is a by-product in the synthesis of cysteine, methionine and arginine, covering around 0.4 mmol/g DCW (Additional file 4). Based on our experimental and literature data, production and re-assimilation of acetate might be over 1 mmol/g DCW at μ = 0.2 h^-1 ^(Text S2 in Additional file 1) which further supports the hypothesis of the necessity for constant acetic acid synthesis.

A similar regulation for overflow metabolism of acetate was posed for *Saccharomyces cerevisiae *by Postma and co-workers: they postulated that acetate accumulation is the result of insufficient acetyl-CoA synthetase activity for the complete functioning of the pyruvate dehydrogenase bypass because of glucose repression of ACS at high growth rates [43]. The hypothesis proposed here is also consistent with the observation that an *acs *mutant of *E. coli *accumulated acetate in chemostat cultures at dilution rate (D) 0.22 h^-1 ^whereas acetate overflow was started in wild-type at a higher D = 0.35 h^-1 ^[28]. Furthermore, it has been shown that over-expression of *acs *[44] and constitutively expressed *acs *together with glyoxylate shunt repressors *iclR *and *fadR *mutant resulted in a significant reduction in acetate accumulation in glucose batch fermentations [28]. Adams and co-workers showed that as a result of micro-evolution, *E. coli *increased acetate consumption capability by over-expressing Acs (not AckA) [45,46], further supporting the connection between Acs activity and acetate accumulation.

As Acs down-regulation is responsible for triggering overflow metabolism and the resulting accumulation of acetate is detrimental to cellular growth, it bears questioning why *E. coli *has not evolved towards maintaining sufficient Acs levels for acetate assimilation in all growth conditions. Growth conditions in *E. coli *native environments are rough as concentrations of utilizable carbon sources including acetate are in the low mg L^-1 ^range and access to nutrients is troublesome [47]. These harsh conditions force *E. coli *to make its metabolism ready for scavenging all possible carbon sources including acetate. However, in nutrient rich laboratory conditions, *E. coli *focuses on anthropic growth [48] and biomass production rate, primarily realized by enhancing readily oxidizable substrate (glucose) uptake kinetics which in turn results in Acs repression through CCR and thus, acetate accumulation [46]. This indicates that an active Acs is not essential for rapid growth for *E. coli*. It seems that maintaining high expression levels of acetate assimilation components (and also other alternative substrates ones) is energetically not favorable at higher growth rates. Moreover, as the space on cell membrane is limited and as *E. coli *achieves more rapid growth probably by increasing the number of glucose transport machinery components on the membrane, using area for alternative substrate transport proteins is not beneficial for faster growth. Interestingly, even in one of its natural environments-urinary tract-where a continuous dilution of acetate occurs, it has been shown that metabolizing acetate to acetyl-CoA by Acs is not essential for normal *E. coli *colonization as PTA-ACKA pathway and maintenance of a proper intracellular acetyl-P concentration are necessary for colonizing murine urinary tract [32].

Based on all the points discussed above, PTA-ACS might function as a futile cycle to provide rapid regulation of acetyl-P concentration in the cell for an active chemotaxis that is vital in natural nutrient-depleted environments, fighting against other organisms (acetate production), pathogenesis, biofilm formation etc. This hypothesis is consistent with the fact that the flagellar assembly and regulation operon (*tar-tap-cheRBYZ*) was more intensively expressed at lower growth rates (Additional file 2) where residual glucose concentration is smaller.

Concerning Acs down-regulation, it is possible that CCR is responsible for its repression as proposed by Treves *et al*. [46] showing the link between ACS expression level and acetate accumulation. In our experiments, it was shown that activation of CCR and repression of Acs take place simultaneously prior to the start of overflow metabolism (Figure 1 and Figure 4). As it is well known that CCR is initiated by the presence of glucose in the medium [49,50], we propose that increasing residual glucose concentration accompanying smooth rise of dilution rate in A-stat triggers Acs down-regulation by CCR. The cAMP-Crp complex is one of the major players in CCR of *E. coli *as cAMP binding to Crp drastically increases its affinity towards activating the promotors of catabolic enzymes, including Acs [6,49,50]. We measured a 1.5-fold decrease in Crp expression with increasing growth rate (Figure 1) that is in agreement with the data in the literature [51]. In addition, when *E. coli *mutant defective in the gene *crp *was cultivated in glucose-limited chemostat at a low D = 0.10 h^-1^, it accumulated acetate whereas the wild-type did not [52]. Furthermore, it exhibited a 34% higher biomass yield relative to the wild-type-this increase might be explained by reduced ATP wasting in the acetate futile cycle, which can be directed to biomass synthesis. Moreover, Khankal *et al*. [53] noted that *E. coli *CRP* mutants that do not require Crp binding to cAMP to activate the expression of catabolic genes showed lowered glucose effect on xylose consumption, 3.6 times higher *acs *expression levels and secreted substantially less acetate in xylitol producing batch fermentations. The connection between cAMP concentration and acetic acid consumption capability, together with the two-phase acetate accumulation profile observed in A-stat and D-stat cultures (Figure 2 and Figure 5) suggests a correlation between increasing residual glucose concentration mediated cAMP-Crp repression and acetate accumulation. Thus, cAMP-Crp dependent regulation of Acs transcribing *acs-yjcH-actP *operon might be a reason for acetate excretion, as also proposed by Veit *et al*. [10]. Our hypothesis of the CCR mediated acetate overflow metabolism is as well in agreement with the fact that rising glucose lowers the intracellular Crp level through the autoregulatory loop of the *crp *gene [54]. However, other mechanisms can also be involved in Acs down-regulation, for example by Cra (Figure 1). Indeed, Sarkar and colleagues have shown that glucose uptake and acetate production rates increased with a decrease of acetate consumption in an *E. coli cra *mutant [55].

What could be the biological relevance of the disruption of the PTA-ACS node? Firstly, decline of the ATP-spending PTA-ACS cycle throughput with increasing growth rate points to possible lower ATP spilling (our model calculations). Secondly, disruption of the PTA-ACS node decreases the energy needed for expression of this cycle's components. As the disruption of PTA-ACS cycle is CCR-mediated, repression of other alternative substrate transport and utilization enzymes by CCR enables to save additional energy. This could all lead to the decrease of ATP production as was indicated by the diminishing TCA cycle fluxes (Figure 2). Hence, it is plausible that cells repress (by CCR) the expression levels of alternative substrate utilization components (including Acs) for making space on the cell membrane for more preferred substrate (glucose) utilization and ATP producing components to achieve faster growth (see above).

Finally, it was demonstrated that highly reproducible A-stat data are well comparable to chemostat at the level of major growth characteristics and transcriptome, hence quasi steady state data from A-stat can be considered steady state representative (Table 1; Figure S1 and Figure S3 in Additional file 1). Furthermore, as shown also by Postma *et al*. for *S. cerevisiae *[43], chemostat is not fully suitable for characterization of dilution rate dependent metabolic transitions, whereas A-stat should be considered an appropriate tool for this. A-stat is especially well suited for the studies of the details of transient metabolism processes. Dynamic behavior of acetate, cAMP etc. with increasing specific growth rate (Figure 2 Figure 3 and Figure S1 in Additional file 1) and change in acetic acid consumption capability in the two-substrate A-stat (Figure S2 in Additional file 1) could be cited as good examples of the latter.

## Conclusion

This study is an excellent example of how a systems biology approach using highly reproducible advanced cultivation methods coupled with multiple 'omics analysis and metabolic modeling allowed to propose a new possible regulation mechanism for overflow metabolism in *E. coli*: acetate overflow is triggered by carbon catabolite repression mediated Acs down-regulation resulting in decreased assimilation of acetate produced by Pta, and disruption of the PTA-ACS node. The practical implications derived from this could lead to better engineering of *E. coli *in overcoming several metabolic obstacles, increasing production yields etc.

## Methods

### Bacterial strain, medium and continuous cultivation conditions

The *E. coli *K12 MG1655 (λ^- ^F^- ^*rph-1Fnr^+^*; Deutsche Sammlung von Mikroorganismen und Zellkulturen, Germany) strain was used in all experiments. Growth and physiological characteristics in accelerostat (A-stat) cultivations were determined using a defined minimal medium as described before by Nahku *et al*. [51], except 4.5 g/L α-(D)-glucose and 100 μl L^-1 ^Antifoam C (Sigma Aldrich, St. Louis, LO) was used. The latter was also used in dilution rate stat (D-stat) experiments as the main cultivation medium. In addition, a second medium was used in D-stat where the main medium was supplemented by acetic acid and prepared as follows: 300 ml medium was withdrawn from the main cultivation medium and supplemented with 3 ml of glacial acetic acid (99.9%). One A-stat experiment (referred to as two-substrate A-stat) was carried out with the same medium as other A-stats, but in addition supplemented with acetic acid (final concentration 5 mM).

The continuous (both A-stat and D-stat) cultivation system consisted of 1.25 L Biobundle bioreactor (Applikon Biotechnology B.V., Schiedam, the Netherlands) controlled by an ADI 1030 biocontroller (Applikon Biotechnology B.V.) and a cultivation control program "BioXpert NT" (Applikon Biotechnology B.V.). The system was equipped with OD, pH, pO_2_, CO_2 _and temperature sensors. The bioreactor was set on a balance whose output was used as the control variable to ensure constant culture volume (300 ± 1 mL). Similarly, the inflow was controlled through measuring the mass of the fresh culture medium.

A-stat cultivation system and control algorithms used are described in more detail in our previous works [24,51,56]. Dilution rate stat (D-stat) is a continuous cultivation method where dilution rate is constant as in a chemostat while an environmental parameter is smoothly changed [24]. The D-stat experiments in this study were carried out with a slight modification: instead of changing an environmental parameter, two different media were used to keep dilution rate constant. After achieving steady state conditions in chemostat using minimal medium supplemented with glucose, addition of the second medium complemented with glucose and acetic acid was started. The feeding rate of the initial medium was decreased at the same time, resulting in constant glucose concentration in the feed. The acetic acid concentration in the bioreactor as a result of inflow has to be determined to enable precise acetic acid consumption/production rate calculation for the bacteria. Hence, increase of acetic acid concentration in bioreactor was calculated and validated in duplicate non-inoculated D-stat test experiments producing an average standard deviation of 1.24 mM between calculated and measured acetic acid concentrations.

All continuous cultivation experiments were carried out at 37°C, pH 7 and under aerobic conditions (air flow rate 150 ml min^-1^) with an agitation speed of 800 rpm. Four A-stat cultivations were performed with acceleration rate (a) 0.01 h^-2^. Duplicate D-stat experiments were performed at dilution rates 0.10; 0.30; 0.505 ± 0.005 h^-1 ^and single experiments at 0.19; 0.24; 0.40; 0.45 h^-1^. The acetic acid addition profile was set to achieve 32 ± 6 mM and 58 ± 5 mM in 7 hours inside the bioreactor for experiments at dilution rates 0.10-0.24 h^-1 ^and 0.30-0.51 h^-1^, respectively. The growth characteristics of the bacteria were calculated on the basis of total volume of medium pumped out from bioreactor (L), biomass (g DCW), organic acid concentrations in culture medium (mM) and CO_2 _concentration in the outflow gas (mM). Formulas were as described in a previous study [24]. It should be noted that the absolute CO_2 _concentrations could be error-prone due to measurement difficulties. However, this does not influence the dynamic pattern of specific CO_2 _production rate (*r*_CO2_) during specific growth rate increase.

### Analytical methods

The concentrations of organic acids (lactate, acetate and formate), ethanol and glucose in the culture medium were determined by HPLC and cellular dry weight (expressed as DCW) as described by Nahku *et al*. [51].

### Protein expression analysis

Refer to Text S1 in Additional file 1 for detailed description. Shortly, protein expression ratios for around 1600 proteins (identified for each growth rate at a > 95% confidence interval in average from 89,303 distinct 2 or more high-confidence peptides) were generated from mass spectrometric spectra by firstly calculating the ratios between continuous cultivation samples at specific growth rates 0.10 ± 0.01 h^-1 ^(chemostat point prior to the start of acceleration in A-stat); 0.20 ± 0.01; 0.26; 0.30 ± 0.01; 0.40 ± 0.00; 0.49 ± 0.01 h^-1 ^and batch sample grown on medium containing ^15^NH_4_Cl as the only source of ammonia. Secondly, the ratios between the mentioned specific growth rates with chemostat point (μ = 0.10 ± 0.01 h^-1^) for two biological replicates were calculated to yield protein expression levels for respective specific growth rates. Protein (and gene) expression measurement results are shown in Additional file 2. Proteomic analysis data is also available at the PRIDE database [57]http://www.ebi.ac.uk/pride under accession numbers 12189-12199 (username: review74613, password: Ge9T48e8). The data was converted using PRIDE Converter http://code.google.com/p/pride-converter[58].

### Gene expression profiling

DNA microarray analysis of 4,321 transcripts was conducted with the Agilent platform using the data from one A-stat cultivation (a = 0.01 h^-2^), and gene expression ratios between specific growth rates 0.21; 0.26; 0.31; 0.36; 0.40; 0.48 h^-1 ^and μ = 0.11 h^-1 ^were calculated. Transcript spot intensities of chemostat sample (sample from D-stat prior to acetic acid addition) from μ = 0.51 h^-1 ^and A-stat μ = 0.48 h^-1 ^were used for the two method's comparison at transcriptome level. Gene (and protein) expression measurement results are shown in Additional file 2. DNA microarray data is also available at NCBI Gene Expression Omnibus (Reference series: GSE23920). The details of the procedure are provided in Text S1 in Additional file 1.

### Metabolome analysis

Sampling was carried out by the rapid centrifugation method. Acquity UPLC (Waters, Milford, MA) together with end-capped HSS C18 T3 1.8 μm, 2.1 × 100 mm column for compound separation coupled to TOF-MS with an electrospray ionization (ESI) source was used for detection (LCT Premiere, Waters). The details of the procedure are provided in Text S1 in Additional file 1.

## Authors' contributions

KV, KA, and RV drafted the manuscript. RN, PL, and LA helped in preparing the manuscript. KV, RN, and PL designed and performed the experiments. KV analysed the experimental data. RN, PL, and LA carried out the 'omics analysis. KV, KA, and RV guided and coordinated the project. All authors read and approved the manuscript.

## Supplementary Material

Additional file 1 Detailed Methods (Text S1); calculation of acetate reconsumption (Text S2); Supplementary Figures S1-S5. Click here for file

Additional file 2 **Growth rate dependent gene (one A-stat) and average protein expression changes of two A-stat experiments with *Escherichia coli *K12 MG1655**. Transcriptome and proteome analysis results, also with standard deviations. Click here for file

Additional file 3 **Gene spot intensities of A-stat at μ = 0.48 h^-1 ^and chemostat at μ = 0.51 h^-1 ^experiments with *Escherichia coli *K12 MG1655**. Data for A-stat and chemostat transcriptome comparison. Click here for file

Additional file 4 **Simplified metabolic flux analysis**. Detailed description of model calculations with simplified metabolic flux analysis. Click here for file
