# Supplementary material for: Systems biology approach reveals that overflow metabolism of acetate in Escherichia coli is triggered by carbon catabolite repression of acetyl-CoA synthetase
Source: BMC Syst Biol. 2010 Dec 1;4:166. doi: 10.1186/1752-0509-4-166 (PMC3014970; doi:10.1186/1752-0509-4-166)
Supplement: Additional file 1 — Detailed Methods (Text S1); calculation of acetate reconsumption (Text S2); Supplementary Figures S1-S5. [file 1752-0509-4-166-S1.PDF]

Additional file 1. Table of Contents

Text S1. Detailed Methods

Text S2. Calculation of acetate reconsumption during CheY acetylation/deacetylation in chemotaxis

Supplementary References.

Figure S1. A-stat reproducibility among three independent *E. coli* cultivations (a = 0.01 h^{-2}) for acetate (Y_{OAc^-}), cAMP (Y_{cAMP}), carbon dioxide (Y_{CO2}) production per biomass and biomass yield (Y_{XS})

Figure S2. Dynamic behaviour of cAMP and acetate consumption capability in *E. coli* two-substrate A-stat cultivation

Figure S3. Correlation between *E. coli* A-stat (μ = 0.48 h^{-1}) and chemostat (μ = 0.51 h^{-1}) log2 gene spot intensities

Figure S4. Correlation of respective log2 protein expression ratios between two *E. coli* A-stat experiments

Figure S5. Correlation of respective log2 gene and average log2 protein expression ratios in *E. coli* A-stat cultures

Text S1. Detailed Methods

Protein expression analysis

Samples intended for proteome analysis were collected from specific growth rates 0.10 ± 0.01 h^{-1} (chemostat point prior to the start of acceleration in A-stat); 0.20 ± 0.01; 0.26; 0.30 ± 0.01; 0.40 ± 0.00; 0.49 ± 0.01 h^{-1}, washed with PBS (0.137 M NaCl, 2.7 mM KCl, 10.0 mM Na_2HPO_4, 1.4 mM KH_2PO_4), flash frozen in liquid nitrogen and stored at -80°C prior to protein extraction. Proteins were extracted in ice-cold SDS-buffer (100mM Tris-HCl (pH 6.5), 1% SDS (w/v), protease inhibitors P8465; Sigma). Cells were disrupted as a result of agitating the suspension with glass-beads at 4°C for 30 minutes. Supernatant was collected after centrifugation (11,500 × *g* at 4°C for 30 min), protein concentration was determined by Ettan *2D Quant Kit* (GE Healthcare, Uppsala, Sweden) and protein samples were stored at -80°C until further analysis.

E. coli cells grown on M9 minimal medium supplemented with $^{15}NH_4Cl$ (98% enrichment; Sigma) as the only nitrogen source for four consecutive times (corresponding to 28 cell doublings) were used as standard sample and added in equal amounts (1:1) to the samples from cultivation experiments. Pooled samples (100 µg) were separated on a 12% acrylamide SDS-PAGE gel (Protean II xi, BioRad, 20x20 cm). The gel was stained with colloidal coomassie, each lane was excised into 10 slices which were cut into 1-2 mm pieces and de-stained by sequential incubations with water and 50% acetonitrile. Subsequently, gel pieces were dehydrated in 100% acetonitrile and reduced in 10 mM DTT/100 mM ammonium bicarbonate solution for 45 min at 56˚C. Alkylation was carried out using 55 mM iodoacetamide/100 mM ammonium bicarbonate solution for 30 min at room temperature in the dark. Gel pieces dehydrated with two volumes of 100% acetonitrile and dried completely in vacuum concentrator (Model 5301, Eppendorf, Cambridgeshire, UK) prior to protease digestion. Trypsin (1:50 enzyme to protein ratio; Promega, Southampton, UK) in 50 mM ammonium bicarbonate was added to gel pieces, digestion was carried out for overnight at 37˚C. Peptides were extracted by sequential incubation with two volumes of 5% FA and 50% acetonitrile. Supernatants were pooled in fresh tubes, concentrated to dryness and kept at -80°C until further analysis.

The digested samples were purified with StageTips [1] and analyzed by LC-MS/MS. Briefly, purified peptides were dissolved in 0.5% formic acid and loaded on self-packed fused silica emitter (150 mm x 0.075 mm; Proxeon, Denmark, packed in-house with Repropur-Sil C18-AQ 3 µm particles (Dr. Maisch, Germany)) using a flow rate of 0.7 µl/min. Peptides were separated on an Agilent 1200 series nanoflow system (Agilent Technologies, Santa Clara, CA) using a 90 or 120 min gradient from 3% to 40% solvent B (solvent A: MilliQ H_20/0.1% formic acid, solvent B: 80% acetonitrile/0.1% formic acid) with a flow-rate of 200 nl/min. Peptides were sprayed directly into LTQ Orbitrap mass-spectrometer (Thermo Electron, Bremen, Germany) operated at 180°C capillary temperature and 2.4 kV spray voltage. One full mass spectra was acquired in profile mode, with mass range from *m/z* 300 to 1900 at resolving power of 60000 (FWHM), following by data-dependent fragmentations on the five most intense peptide ions acquired in centroid mode in the linear ion trap. Each fragmented ion was dynamically excluded for 60 s.
Peak lists for database searches were produced with Raw2MSM version 1.7 [2] selecting top six peaks for 100 Da. MSM files for the same sample were concatenated to generate a single large peak list file with MultiRawPrepare.pl script (http://msquant.alwaysdata.net) and subsequently searched with the Mascot 2.2 search engine (Matrix Science, London, UK) against the custom-

made *E. coli* protein database, that included *E. coli* K-12 MG1655 protein sequence database downloaded from EcoGene 2.0 (http://ecogene.org) and most commonly observed contaminant sequences, such as proteases, keratins etc.. Search parameters were as follows: one missed trypsin cleavage; carbamidomethyl was set as a fixed modification; oxidation (M) as variable modification; precursor ion mass tolerance was 5 ppm and fragment mass tolerance 0.6 Da; ^{15}N metabolic labelling was selected as the quantification method and the Mascot built-in decoy database option was used to estimate the false discovery rate (FDR) for peptides. A Mascot peptide cut-off score of 10 was used for further data analysis, corresponding to a false discovery rate less than 0.65%. Quantification of $^{15}N/^{14}N$ ratios was performed using MSQuant [3]. Quantification was based only on proteins that were identified with at least two peptides with Mascot peptide score > 20. All quantified spectra were manually validated to remove borderline hits and erroneous quantifications. Quantification data were normalized on the median of all measured $^{15}N/^{14}N$ ratios. Two biological replicates were compared at specific growth rates 0.20 ± 0.01; 0.26; 0.30 ± 0.01; 0.40 ± 0.00; 0.49 ± 0.01 h^{-1} with sample at $\mu = 0.10 \pm 0.01$ h^{-1} (chemostat point after stabilizing the culture in A-stat). Protein (and gene) expression measurement results are shown in Additional file 2. Proteomic analysis data is also available at the PRIDE database [4] (www.ebi.ac.uk/pride) under accession numbers 12189-12199 (username: review74613, password: Ge9T48e8). The data was converted using PRIDE Converter (http://code.google.com/p/pride-converter) [5].

Gene expression profiling

Agilent's DNA microarrays were designed in eArray web portal in 8 X 15K format containing 3 probes per target (https://earray.chem.agilent.com/earray/). Target sequences for 4,321 genes were downloaded from Kyoto Encyclopedia of Genes and Genomes (ftp://ftp.genome.jp/pub/kegg/genes/organisms/eco/e.coli.nuc).

RNA degradation was halted, total RNA extracted, cDNA synthesized and labeled as described previously [6], with minor modification: 15 µg of total RNA was used for cDNA synthesis. Hybridization, slide washing and scanning was performed using standard Agilent's reagents and hardware (www.chem.agilent.com). Gene expression data was analyzed as described before [6], except global lowess normalization was used. In addition, genes left with only one spot after removing spots with signal-to-noise ratio less than three were considered unreliable and thus, not included in analysis. Average log2 gene expression changes and spot intensities are shown only for genes with relative standard deviation smaller than 20% among its multiple spots. Gene (and protein) expression measurement results are shown in Additional file 2. DNA microarray data is also available at NCBI Gene Expression Omnibus (Reference series: GSE23920).

Metabolome analysis

Sampling was carried out by the rapid centrifugation method [7] where 1 ml of culture broth was transferred into an 1.5 ml eppendorf and centrifuged for 16 s at 14,000 × *g* in a tabletop centrifuge. The supernatant was decanted and the pellet was immediately frozen in liquid nitrogen (transfer of pellets from bioreactor to liquid nitrogen did not take longer than 30 s). At least triplicate samples from the same time point were analyzed. Metabolites were extracted using 60% (vol/vol) ethanol buffered with 10 mM ammonium acetate at pH 7.2. Pellets were extracted three times at 70°C for 1.5 min by adding 0.5 ml of extraction solution and shaking in Thermomixer (Eppendorf, Hamburg, Germany). Cell debris was separated by centrifugation (14,000 × *g* for 1 min) and the supernatant was pooled on ice.

Samples were dried to complete dryness and then re-suspended in 50 µL of milliQ H_2O by vortexing. Acquity UPLC (Waters) together with end-capped HSS C18 T3 1.8 µm, 2.1 x 100 mm column was used for compound separation. For detection, TOF-MS with an electrospray ionization (ESI) source was used (LCT Premiere, Waters) with the following parameters: negative ionization mode, desolvation gas 700 L/h and 200°C, capillary voltage 2500 V, sample cone 30 V. Peak detection and integration was performed with MassLynx Software (Waters). milliQ H_2O with an electrical resistance of greater than 18.6 MΩ and organic solvents of Chromasolv LC/MS grade (Sigma-Aldrich) were used for preparation of eluents. The ion-pairing LC method was adapted from a method that was published by Luo *et al.* [8]. The mobile phase was composed of eluent A (aqueous solution of 10 mM tributylamine and 15 mM acetic acid in 5% of methanol) and eluent B (methanol); the gradient profile was as follows: t) 0 min, 0% B; t) 1.6 min, 20% B; t) 7.5 min, 40% B; t) 9.5 min, 100% B. The column was washed at the end of the gradient with 100% eluent B for 1.5 min (1.3 column volumes) and equilibrated before each injection for 4 min (3.5 column volumes). At all times, the flow rate was 400 µL/min, the column temperature was controlled at 40°C, and the injection volume was 1.1 µL.

Text S2. Calculation of acetate reconsumption during CheY acetylation/deacetylation in chemotaxis

CheY acetylation/deacetylation plays a role in switching of flagellar movement from clockwise to counter clockwise to change the direction of cell movements [9]. It has been shown that CheY concentraion in the cell is around 8 mM and acetylated at 3-9 sites [9]. Taking the mass for one cell 10^{-12} g and 30% of that is dry matter, then 80-240 nmol/g DCW acetate is needed for CheY acetylation. Taking flagellar switching from clockwise to counter-clockwise once per second [10] and biomass production (X =1.8 g DCW/L) at the specific growth rate 0.2 h^{-1} is 0.11 g/h, then around 0.3-0.9 mmol/g DCW acetate is cycling continuously.

Supplementary References

1. Rappsilber J, Mann M, Ishihama Y: **Protocol for micro-purification, enrichment, pre-fractionation and storage of peptides for proteomics using StageTips.** *Nat Prot* 2007, **2**:1896-1906.
2. Olsen JV, de Godoy LM, Li G, Macek B, Mortensen P, Pesch R, Makarov A, Lange O, Horning S, Mann M: **Parts per million mass accuracy on an Orbitrap mass spectrometer via lock mass injection into a C-trap.** *Mol Cell Proteomics* 2005, **4**:2010-2021.
3. Mortensen P, Gouw JW, Olsen JV, Ong SE, Rigbolt KT, Bunkenborg J, Cox J, Foster LJ, Heck AJ, Blagoev B, Andersen JS, Mann M: **MSQuant, an Open Source Platform for Mass Spectrometry-Based Quantitative Proteomics research articles.** *J Proteome Res* 2010, **9**:393-403.
4. Martens L, Hermjakob H, Jones P, Adamski M, Taylor C, States D, Gevaert K, Vandekerckhove J, Apweiler R: **PRIDE: the proteomics identifications database.** *Proteomics* 2005, **5**:3537-3545.
5. Barsnes H, Vizcaíno JA, Eidhammer I, Martens L: **PRIDE Converter: making proteomics data-sharing easy.** *Nat Biotechnol* 2009, **27**:598-599.
6. Nahku R, Valgepea K, Lahtvee PJ, Erm S, Abner K, Adamberg K, Vilu R: **Specific growth rate dependent transcriptome profiling of *Escherichia coli* K12 MG1655 in accelerostat cultures.** *J Biotechnol* 2010, **145**:60-65.
7. Kleijn RJ, Buescher JM, Le Chat L, Jules M, Aymerich S, Sauer U: **Metabolic fluxes during strong carbon catabolite repression by malate in *Bacillus subtilis*.** *J Biol Chem* 2009, **285**:1587-1596.
8. Luo B, Groenke K, Takors R, Wandrey C, Oldiges M: **Simultaneous determination of multiple intracellular metabolites in glycolysis, pentose phosphate pathway and tricarboxylic acid cycle by liquid chromatography–mass spectrometry.** *J Chromotgr A* 2007, **1147**:153-164.
9. Barak R, Eisenbach M: **Correlation between phosphorylation of the chemotaxis protein CheY and its activity at the flagellar motor.** *Biochemistry* 1992, **31**:1821-1826
10. Blair D: **Flagellar movement driven by proton translocation.** *FEBS Lett* 2003, **545**:86-95.

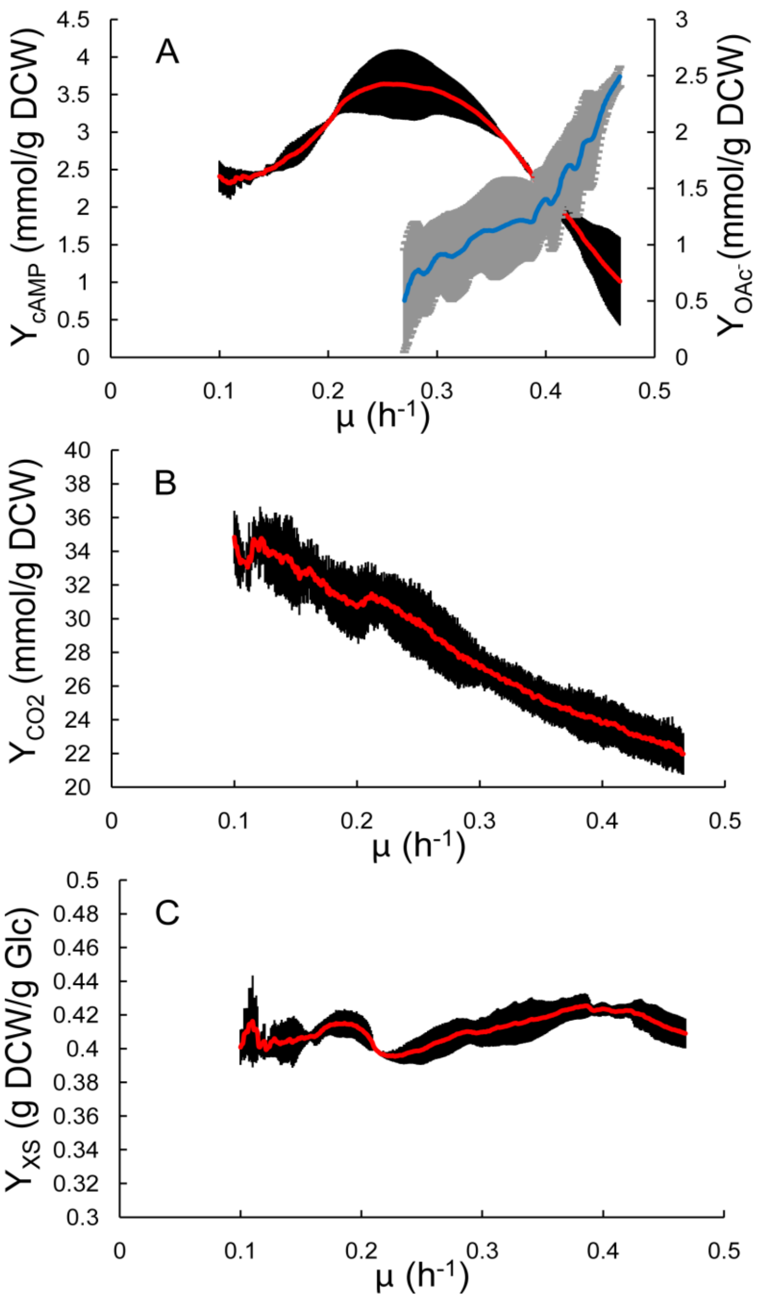

Figure S1

A-stat reproducibility among three independent *E. coli* cultivations (a = 0.01 h^{-2}) for acetate (Y_{OAc}-), cAMP (Y_{cAMP}), carbon dioxide (Y_{CO2}) production per biomass and biomass yield (Y_{XS}). μ, specific growth rate (h^{-1}). Y_{XS} is calculated as biomass formed (g dry cellular weight (DCW)) for 1 g of glucose consumed (g Glc). (*A*) Red, blue lines indicate average and black, grey areas standard deviation of Y_{cAMP} and Y_{OAc}-, respectively. Y_{OAc}- values start from the first point when acetate was detected. (*B*), (*C*) Red line indicates average and black area standard deviation. Average relative standard deviations within the studied specific growth rate range were as follows: Y_{XS} = 2.0%; Y_{CO2} =5.6%; Y_{cAMP} =9.1%. Start of vertical axes was chosen for better visualization.

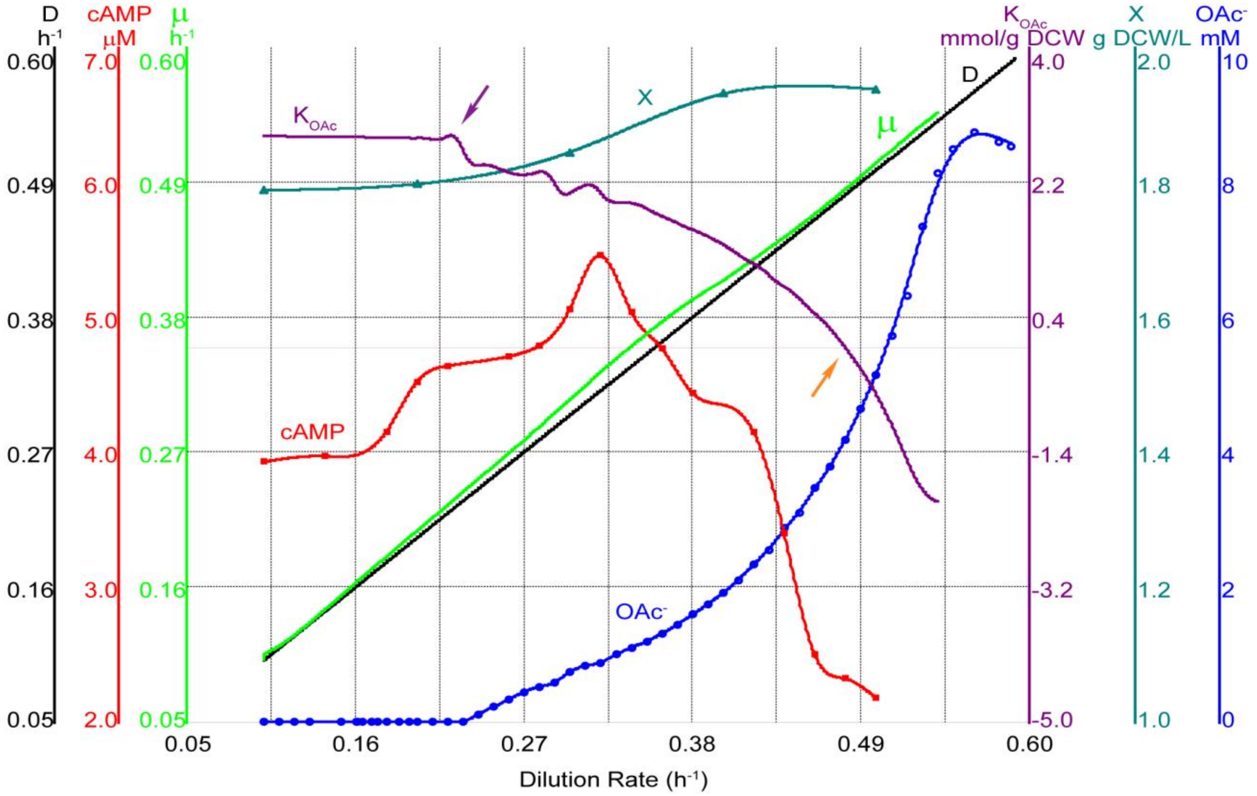

Figure S2

Dynamic behaviour of cAMP and acetate consumption capability in *E. coli* two-substrate A-stat cultivation. D, dilution rate (h^{-1}); X, biomass concentration (g dry cellular weight (DCW)/L); μ, specific growth rate (h^{-1}); OAc^-, acetate concentration (mM); K_{OAc}-, acetic acid consumption per biomass (mmol/g DCW); cAMP, cyclic AMP concentration (μM). Purple arrow denotes change in acetic acid consumption capability whereas orange arrow depicts complete abolishment of acetic acid consumption capability.

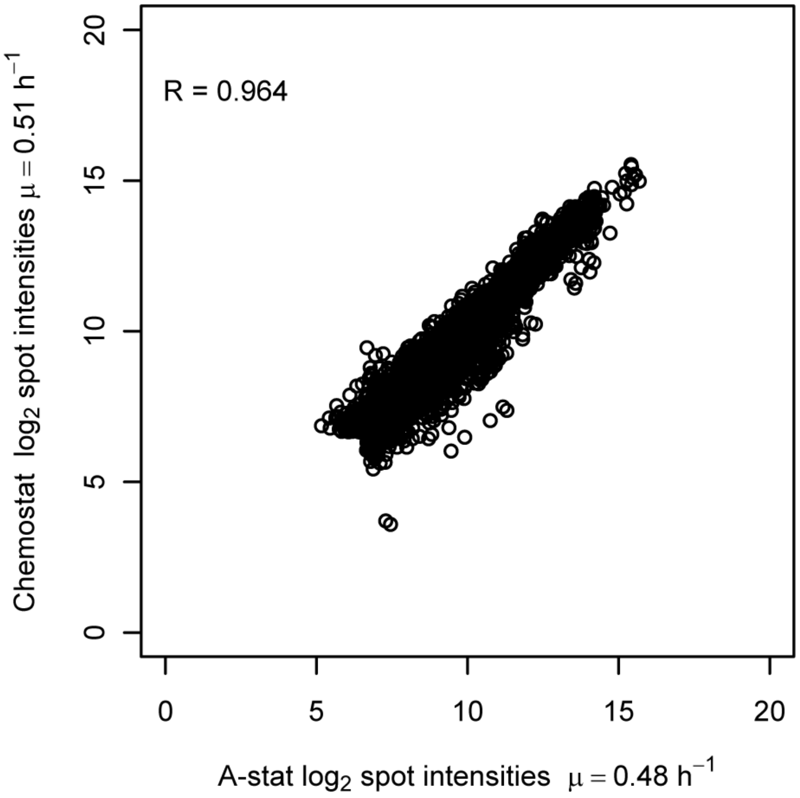

Figure S3

Correlation between *E. coli* A-stat (μ = 0.48 h^{-1}) and chemostat (μ = 0.51 h^{-1}) log2 gene spot intensities. R, Pearson correlation coefficient. Genes with relative standard deviation smaller than 20% among its multiple spots were taken into account for Pearson correlation coefficient calculation.

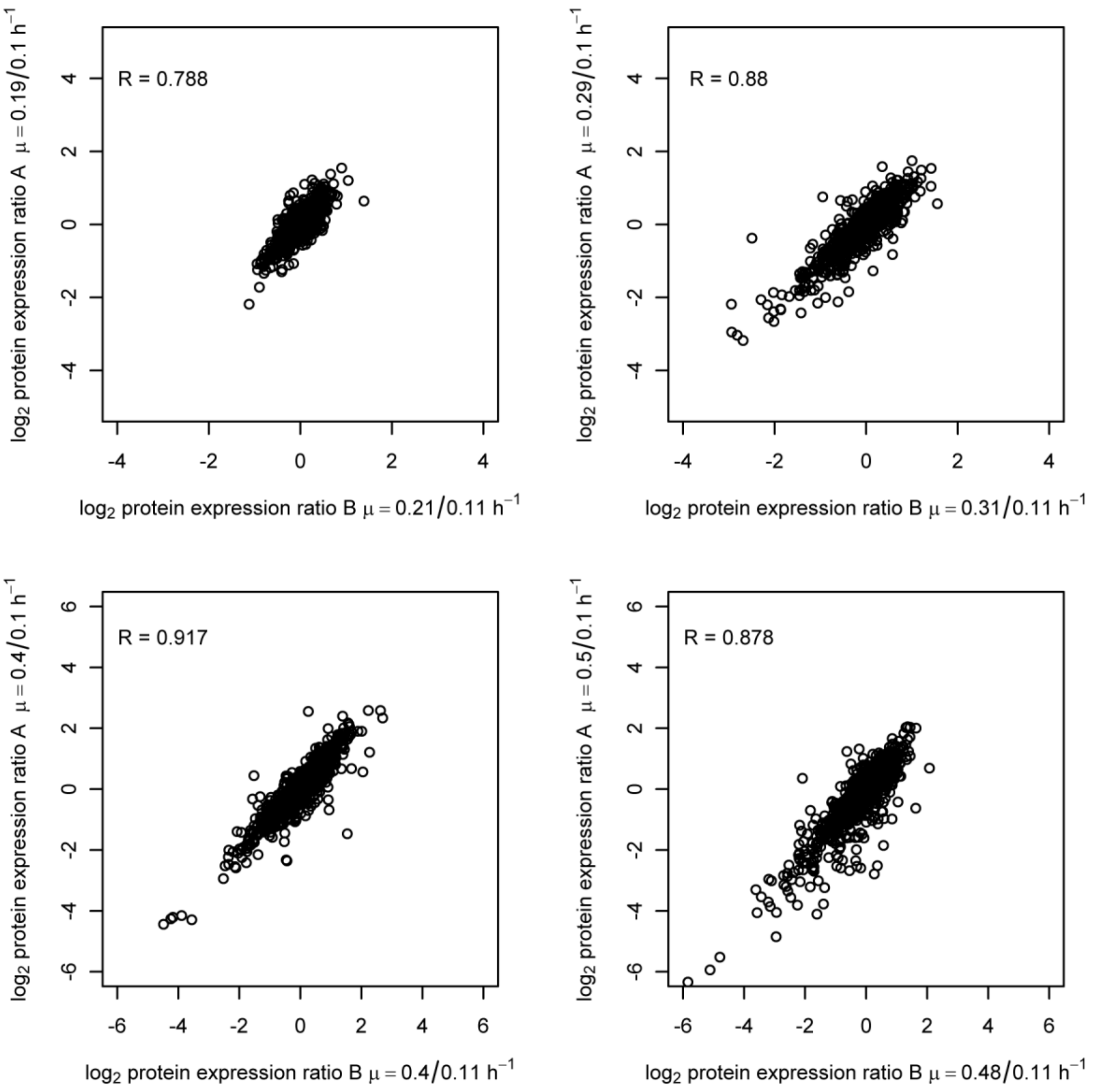

Figure S4

Correlation of respective log2 protein expression ratios between two *E. coli* A-stat experiments. μ, specific growth rate (h^{-1}). R, Pearson correlation coefficient. A and B represent duplicate experiments, respectively.

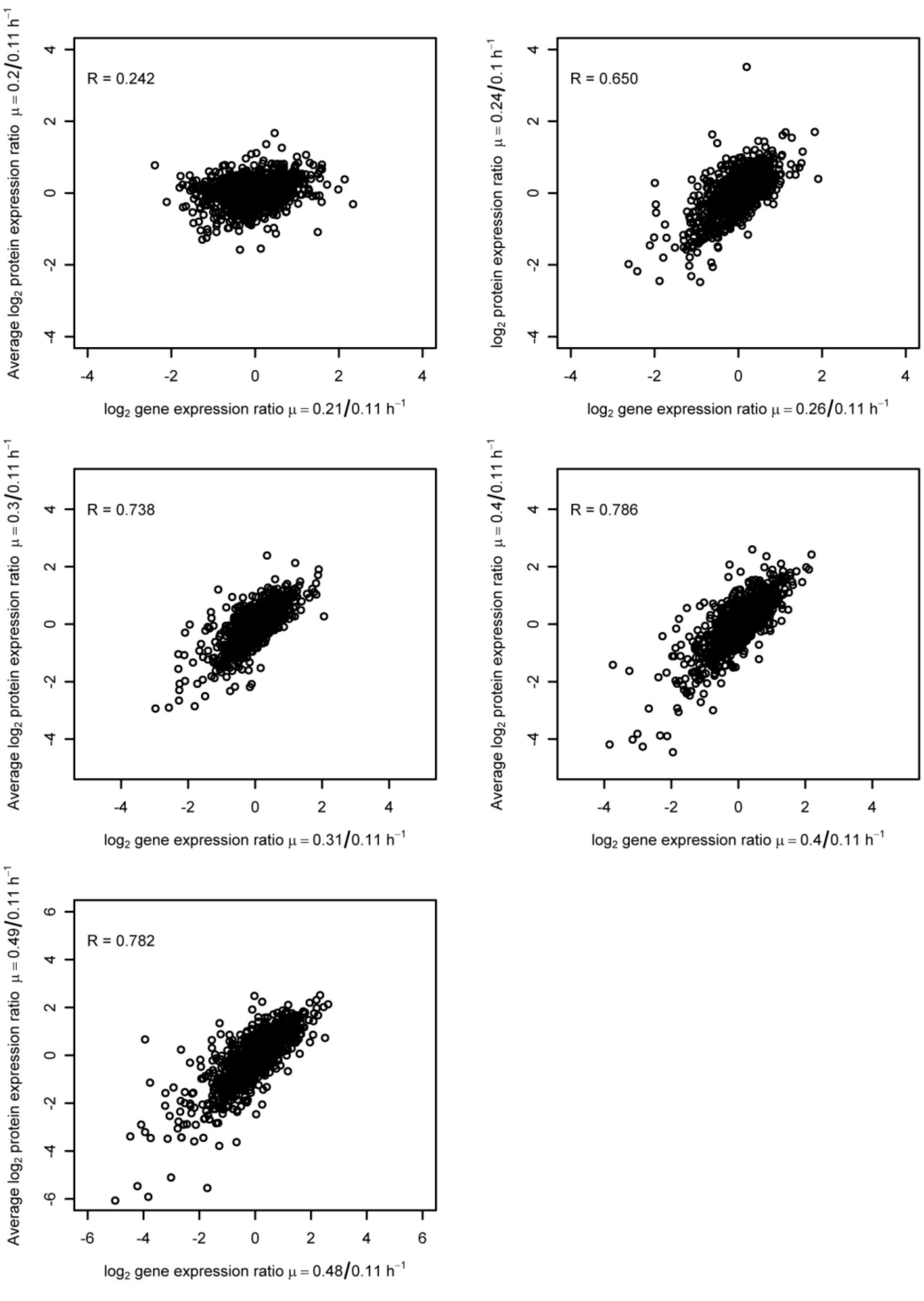

Figure S5

Correlation of respective log2 gene and average log2 protein expression ratios in *E. coli* A-stat cultures. μ, specific growth rate (h^{-1}). R, Pearson correlation coefficient. Average log2 protein expression ratios represent average from two independent experiments. Proteome analysis at μ = 0.24 h^{-1} was carried out in one experiment. Genes with relative standard deviation smaller than 20% among its multiple spots were included for analysis.
