# Supplementary material for: Systems biology approach reveals that overflow metabolism of acetate in Escherichia coli is triggered by carbon catabolite repression of acetyl-CoA synthetase
Source: BMC Syst Biol. 2010 Dec 1;4:166. doi: 10.1186/1752-0509-4-166 (PMC3014970; doi:10.1186/1752-0509-4-166)
Supplement: Additional file 4 — Simplified metabolic flux analysis. Detailed description of model calculations with simplified metabolic flux analysis. [file 1752-0509-4-166-S4.PDF]

Additional file 4. Simplified metabolic flux analysis

Simplified metabolic network scheme of *Escherichia coli* K12 MG1655 was reconstructed taking into account main metabolic pathways (glycolysis, pentose phosphate pathway and TCA cycle) which involved only fluxes (reactions) between branching points (metabolites) whereas linear pathway chains were lumped together (Fig. S6). Fully determined and calculable stoichiometric matrix consists of 18 metabolites and 38 fluxes (18 dependent fluxes, 1 measured inflow, two ouflows and 17 calculated fluxes based on biomass composition and stoichiometries of biosynthetic pathways). Cofactors ATP, NADPH and NADH were considered in calculations. Metabolic flux analysis (MFA) method was implemented for the selected simplified network scheme using MS Excel 2007 linear algebra routines for the calculation of intracellular steady-state flux distributions. MFA is based on the assumption of steady state in intracellular concentrations of metabolites and it has been applied mostly for the analysis of physiological states obtained from batch (exponential phase) and chemostat at fixed dilution rate. However, quasi steady state growth can be also achieved in changing conditions using low acceleration rates of environmental parameters. Quasi steady state is defined as the growth state until μ_{crit} is reached *i.e.* specific growth rate (μ) differs from dilution rate (D) more than 5 %. It should be emphasized that until achieving μ_{crit}, the A-stat quasi steady state culture is steady state representative as shown by this study. Calculations were constrained by experimentally determined data from three A-stat experiments at a=0.01 h^{-1} and calculated flux patterns had units μmol/g dry cellular weight (DCW).

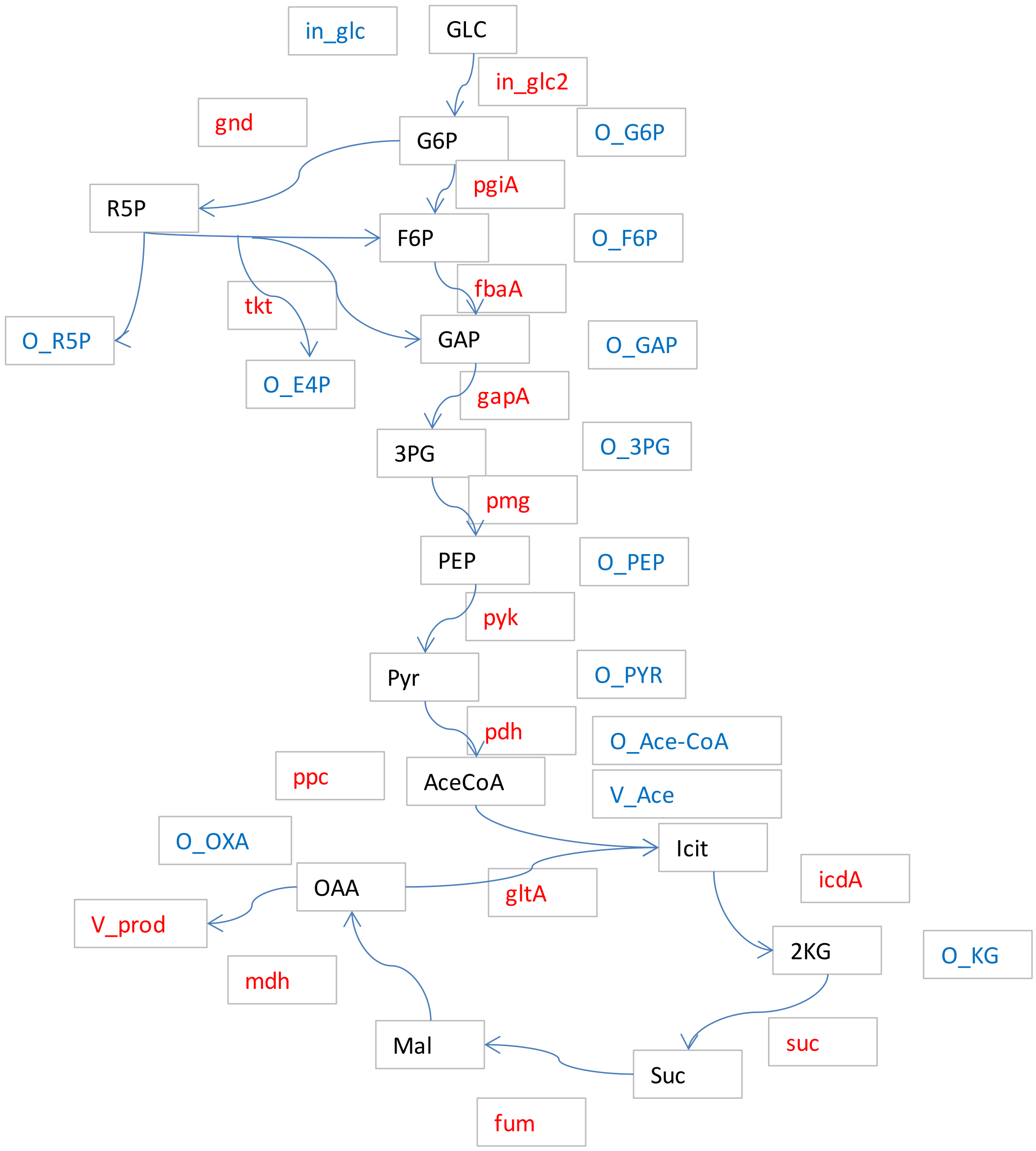

Figure S6

Simplified metabolic network. Metabolites are indicated in black, calculated fluxes in red and measured fluxes in blue. G6P, glucose-6-phosphate; F6P, fructose-6-phosphate; R5P, ribose-5-phosphate; GAP, glyceraldehydephosphate; 3PG, 3-phosphoglycerate; PEP, phosphoenolpyruvate; PYR, pyruvate; Ace-CoA, acetyl-CoA; SUC, succinate; ICIT, isocitrate; 2KG, 2-oxoglutarate; MAL, malate; OXA, oxaloacetate.

18 dependent fluxes were:

1. In_glc2: Glc_ext + ATP = G6P
2. gnd: G6P = 2NADPH + R5P + CO2
3. tkt: 3R5P = 2F6P + GAP
4. pgiA: G6P = F6P
5. fbaA: F6P + ATP = 2GAP
6. gapA: GAP = NADH + ATP + 3PG
7. pmg: 3PG = PEP
8. pyk: PEP = ATP + PYR
9. pdh: PYR = NADH + Ace-CoA + CO2
10. gltA: Ace-CoA + OXA = ICIT
11. icdA: ICIT = NADPH + 2KG + CO2

12. suc: 2KG = ATP + NADH + SUC + CO2
13. fum: SUC = 2ATP + MAL
14. mdh: MAL = NADH + OXA
15. ppc: PEP + CO2 = OXA
16. vNADH: NADH = 2 ATP
17. ngATP: ATP =
18. vprod : OXA =

Note: ngATP characterizes non-growth associated ATP expenditure if biomass components are synthesized once. vNADH characterizes ATP production in respiratory chain if P/O = 2. Vprod characterizes carbon outflow that was not identified experimentally.

3 measured fluxes were:

1. in_GLC: = GLC_ext
2. Vace: Ace-CoA = ATP
3. VCO2: CO2 =

17 calculated fluxes were:

1. O1: G6P =
2. O2: F6P =
3. O3: R5P =
4. O4: 2R5P = F6P
5. O5: GAP =
6. O6: 3PG =
7. O7: PEP =
8. O8: PYR =
9. O9: Ace-CoA =
10. O10: OXA =
11. O11: 2KG =
12. Onadh: = NADH
13. Onadph: NADPH =
14. Osynth: ATP =
15. Opolym: ATP =
16. mATP: ATP =
17. acetrans: 2ATP = AceCoA

Note: acetrans characterizes conversion of acetate (produced during arginine, cysteine and methionine synthesis) to Ace-CoA by ACS.

O-fluxes were calculated based on biomass monomer composition and reaction stoichiometries from central metabolites to monomers (20 amino acids, 8 nucleotides, 6 fatty acids and 3 mono-saccharides, see Table S1). Amino acids (using AccQTaq Ultra pre-column derivatization kit and UPLC (Waters Corp.) according to the manufacturer's instructions from hydrolyzed culture samples), fatty acids (using saponification by KOH and fatty acid quantification by UPLC as in Špitsmeister *et al.*[1]) and total RNA (using Qiagen RNA quantification kit) were experimentally measured from biomass. DNA and ash content in biomass was taken from Neidhardt *et al*, (1987) [2] and residual water in dry biomass was estimated as 8 %. Polysaccharide content was calculated as residual of above mentioned components (Table S1).
Model calculations are given in Table S2, Figures S6 and S7.

Table S1
Biomass monomer composition at different specific growth rates (µmol/g DCW).

µ, h^{-1}	0.1	0.2	0.3	0.4	0.5
Glucose	1328	1162	999	842	692
NAG*	42	42	42	41	40
NAM*	42	42	42	41	40
dAMP	25	25	25	25	25
dTMP	25	25	25	25	25
dCMP	25	25	25	25	25
dGMP	25	25	25	25	25
AMP	51	73	95	117	139
UMP	42	60	78	96	114
CMP	39	55	72	89	106
GMP	62	89	117	144	171
glycerol	126	125	123	122	121
C14	21	21	21	20	20
C16	69	60	56	56	61
C16:1	48	57	63	66	66
C18:1	30	43	52	56	56
C17	78	67	56	45	34
C19	4	3	2	2	1
Ala	473	472	471	469	468
Arg	226	232	239	245	252
Asp+Asn	372	372	372	372	371
Cys	23	23	22	22	22
Glu+Gln	279	281	283	285	286
Gly	393	397	400	403	407
His	88	87	87	87	86
Ile	217	217	218	218	218
Leu	371	373	375	377	379
Lys	243	249	255	261	267
Met	105	105	106	106	106
Phe	160	159	157	156	154
Pro	171	172	173	175	176
Ser	223	222	220	218	217
Thr	238	238	237	237	236
Trp	17	18	18	18	19
Tyr	148	148	148	148	148
Val	288	292	296	300	304

*NAG - N-acetylglucosamine, NAM - N-acetylmuramic acid

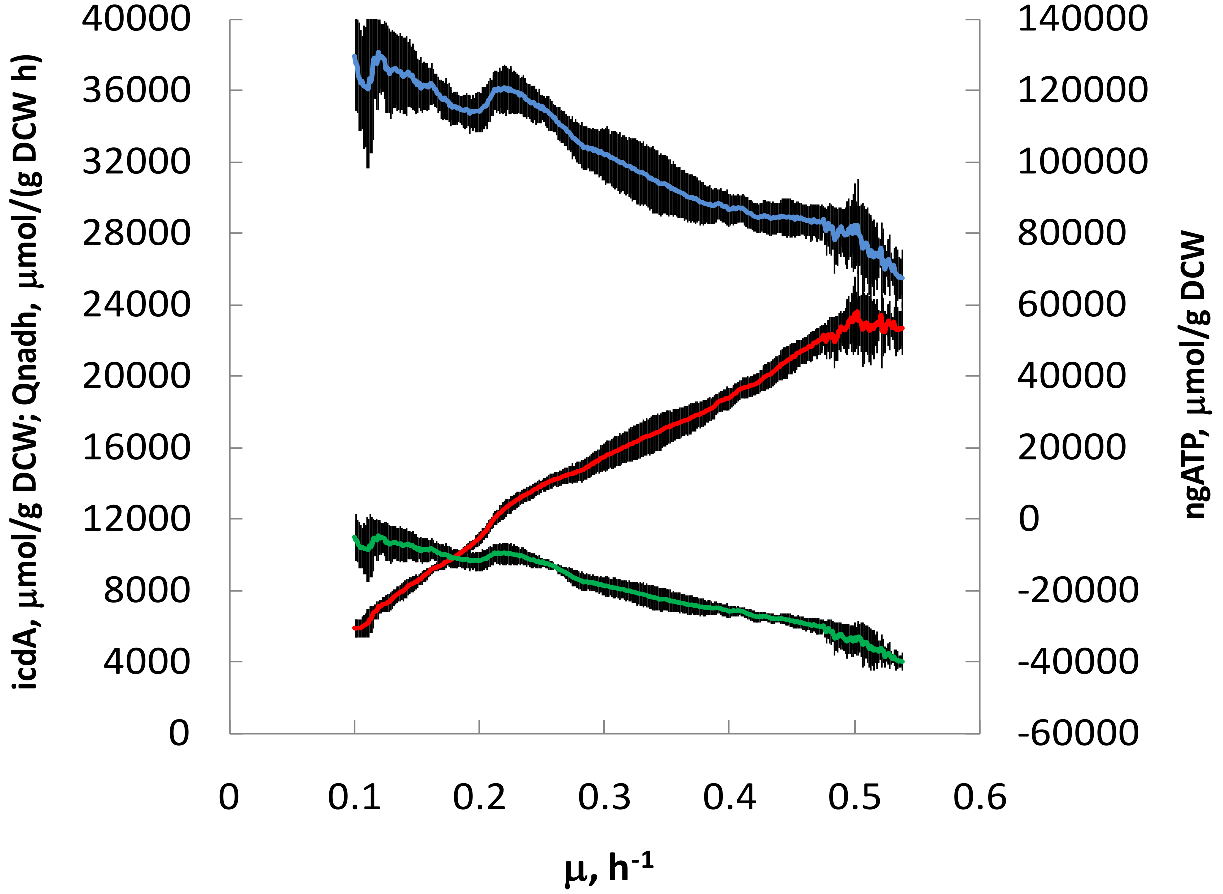

Figure S7

Model calculations for non-growth associated ATP production (ngATP), representative TCA flux (icdA) and specific NADH production rate (Qnadh) in *E. coli* A-stat cultures. ngATP is shown in blue; icdA in green; Qnadh in red. Black area represents standard deviation.

Table S2
Summary of calculated fluxes in *E. coli* A-stat cultures at different specific growth rates (µmol/g DCW).

	$\mu = 0.1\ h^{-1}$		$\mu = 0.2\ h^{-1}$		$\mu = 0.3\ h^{-1}$		$\mu = 0.4\ h^{-1}$		$\mu = 0.5\ h^{-1}$	
	average	stdev	average	stdev	Average	stdev	average	stdev	average	stdev
in_glc2	13666	559	13525	278	13552	287	13120	126	13993	533
pgiA	11016	1242	10459	360	9915	578	8858	272	9076	773
fbaA	11449	791	11225	272	11119	395	10523	179	11223	602
gapA	22867	1359	22589	524	22598	698	21636	311	23279	1137
pmg	22057	1354	21727	536	21680	687	20674	306	22267	1132
pyk	15393	1366	14433	505	13986	846	13009	507	13851	1030
pdh	13066	1363	12053	506	11549	835	10526	501	11318	1024
Vnadh	58118	5444	54966	1672	51894	2630	47120	1362	46556	3178
ppc	6030	199	6663	678	7067	164	7040	206	7794	111
gltA	10671	1362	9670	505	8306	534	6850	289	5283	735
icdA	10671	1362	9670	505	8306	534	6850	289	5283	735
ngATP	125453	16270	114206	5365	102495	7502	86784	4004	80915	9013
Vprod	4040	190	4621	687	4970	172	4900	215	5604	106
gnd	1350	675	1936	254	2683	256	3445	145	4247	367
suc	9994	1362	8983	505	7609	532	6144	289	4568	734
fum	9994	1362	8983	505	7609	532	6144	289	4568	734
tkt	95	227	263	84	482	92	712	48	953	125
Mdh	9994	1362	8983	505	7609	532	6144	289	4568	734

Supplementary References

1. Špitsmeister M, Adamberg K, Vilu R: **UPLC/MS based method for quantitative determination of fatty acid composition in Gram-negative and Gram-positive bacteria.** *J Microbiol Methods* 2010, **82**:288-95.
2. Neidhardt FC: ***Escherichia coli* and *Salmonella typhimurium*: Cellular and molecular biology**. Edited by Neidhardt FC. Washington, D.C: American Society for Microbiology; 1987:4.
